# Dental tray adhesives and their role as potential transmission medium for microorganisms

**DOI:** 10.1002/cre2.432

**Published:** 2021-05-06

**Authors:** Oliver Schierz, Henrik Müller, Catalina Suzana Stingu, Sebastian Hahnel, Angelika Rauch

**Affiliations:** ^1^ Department of Prosthodontics and Materials Science University of Leipzig Leipzig Germany; ^2^ Private Dental Practice Bitburg Germany; ^3^ Institute for Medical Microbiology and Epidemiology of Infectious Diseases University Hospital of Leipzig Leipzig Germany

**Keywords:** antimicrobial properties, cross‐contamination, impression tray adhesive, mass spectrometry

## Abstract

**Objectives:**

This study aimed to evaluate the possible ability of dental impression tray adhesives to serve as a transmission medium for bacteria and fungi when reusable adhesive applicators are utilized.

**Materials and methods:**

Ten flasks with tray adhesive were monitored over a period of 12 weeks during clinical use for contamination with bacteria or fungi. Adhesive fluid samples were cultivated on eight different culture media. All grown colonies were identified by using mass spectrometry (MALDI‐TOF). Isolates without reliable identification were either identified by Rapid ID 32 API‐STREP V3.0 or by sequencing the 16S rRNA genes.

**Results:**

After 4 weeks, bacterial growth was detected on chocolate blood agar plates in five different samples. The bacterial species were identified as *Staphylococcus warnerii*, *Staphylococcus epidermidis*, *Staphylococcus pasteuri*, *Ralstonia insidiosa*, and *Alloiococcus otitidis*. After 8 weeks Streptococcus oralis grew on a blood agar plate. In all samples, no fungi were identified.

**Conclusions:**

The disinfectant component of the tested tray adhesive seems to be effective. However, some bacteria survived in the flask for a clinically relevant time, which might result in a potential transmission to a new host.

## 1 INTRODUCTION

For the safety of both patients and medical health care providers, infection control is a major concern in dentistry (Cristina et al., 2009; Sabino‐Silva et al., 2020). The current COVID crisis increased the consciousness and awareness for the transmission of microorganisms. The oral microbiome is featured by a high variation and a vast diversity and contains numerous bacteria and fungi species (Renson et al., 2019).

**FIGURE 1:**
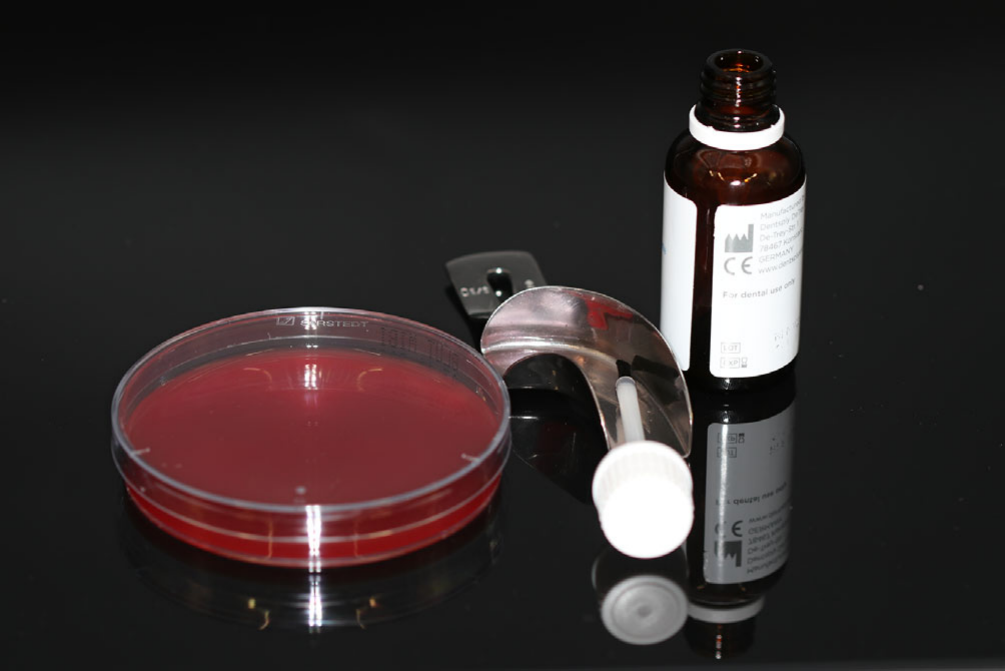
Agar plate, dental tray, reusable brush, and adhesive flask

Impression tray adhesives are commonly delivered in glass flasks featuring a plastic cap with a fixed brush (Figure 1). Impression tray adhesives are commonly used to retain dental impression material fixed to the impression tray, preventing deformation of the impression by partial detachment. When the brush is used on an impression tray that was not properly disinfected subsequent to the try‐in procedures, both the brush and the adhesive within the flask might be contaminated by the oral microorganisms. Consequently, there is a plausible risk for transmitting oral bacteria and fungi from patient to patient. As an alternative approach, the application of the tray adhesives with a spray would be safer from a hygienic standpoint, but aerosol formation and a risk of combustion when applied near open fire are disadvantageous. Another alternative would be the use of extra vessels or single dose blisters with disposable applicators, causing higher costs and increase waste. For infection control, volatile substances like isopropanol or ethyl acetate are added to the dental tray adhesives.

A previous in‐vitro study revealed no relevant growth inhibition of oral bacteria induced by these adhesives. Alginate adhesive showed the most intense bacterial growth in this study (Bensel et al., 2013). However, due to the study design, it is possible that the disinfectant component of the adhesive did not operate effectively and volatized before the bacteria have been applied. Another study artificially contaminated suspensions of dental adhesives with three test organisms (*Staphylococcus aureus*, *Salmonella* Choleraesuis, *Pseudomonas aeruginosa*). In contrast to the other investigation, a significant inhibition of oral bacterial growth after 24 h was observed (Herman, 1993). Based on the authors' knowledge, no studies have ever investigated the bacterial and fungal contamination of the liquid within the glass flasks of dental tray adhesives in clinical dental practice. The purpose of this explorative study was to measure the contamination of these adhesives under clinical conditions and to establish a qualitative screening for bacteria and fungi. The null hypothesis stated that no microorganisms can be cultivated from the liquid within the glass flasks tray adhesives.

## 2 MATERIALS AND METHODS

Ten flasks, each containing 30 mL tray adhesive fluid for alginate (Fix Adhesive Liquid, charge 1,408,004,128, Dentsply DeTrey GmbH, Konstanz, Germany), were monitored over 12 weeks to detect signs of contamination during running clinical undergraduate student courses and conventional prosthodontic treatment operated by qualified dentists. Beside routine hygiene instructions in introductory courses prior to the clinical student courses neither students nor personal were sensitized by additional hygienic instructions to prevent cross‐contamination using tray adhesives. The adhesive was composed as follows: diethylentriamin‐polymer as dissolvent, isoropanol as desinfectant, xylol (aromatic hydrocarbons), and erythrosin as coloring agent.

**FIGURE 2:**
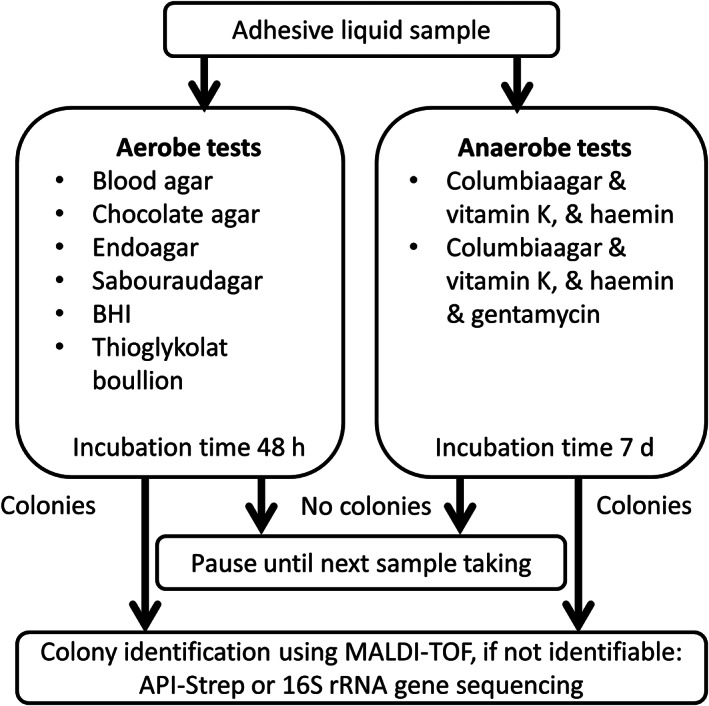
Examination procedure of the samples; MALDI‐TOF = laser‐desorption‐ionization‐time‐of‐flight mass spectrometry

**FIGURE 3:**
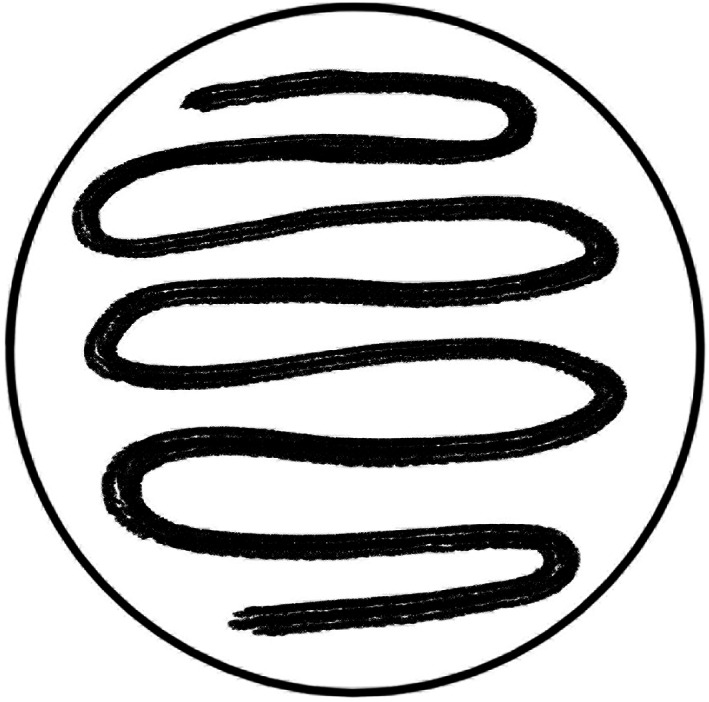
Sample spreading path over the medium for inoculation using the cap‐attached brush

All microbiological analyses were performed by the same examiner. For sample taking, 10 flasks were collected five times, one from each assigned examination room after business hours, which was after two, four, six, eight, and 12 weeks. Once culture media were inoculated by the reusable brush, flasks were relocated to their original place of storage for subsequent clinical use. Eight different culture media were used (Figure 2). Liquid samples were applied on all media in a biological workbench (HeraSafe KS Class II Workbench, Thermo Fisher Scientific, Dreieich, Germany). Therefore the adhesive attached to the brush was systematically spread out all over the whole culture medium (Figure 3). The media were incubated aerobically at 37°C in a CO_2_‐Incubator (Heracell 150i CO_2_ Incubator, Thermo Fisher Scientific, Dreieich, Germany) with approximately 5% CO_2_ for 48 h. Cultures on Columbia agars were anaerobically incubated (Whitley MG 1000, anaerobic workstation, Meintrup Laborgeräte GmbH, Lähden, Germany) at 37°C for 7 days. Growing colonies were identified by using matrix–assisted laser–desorption–ionization‐time‐of‐flight mass spectrometry (MALDI‐TOF; VITEK® MS, bioMérieux, Lyon, FranceIsolates not reliably identified by MALDI‐TOF, were subsequently differentiated by rapid ID 32 API‐STREP V3.0 (bioMérieux) or by 16S rRNA genes sequencing (Clark et al., 2013; Petti et al., 2005). Descriptive analyses were performed by using statistical software package STATA (Stata Statistical Software: Release 15.1. StataCorp LP, College Station, TX).

## 3 RESULTS

After two, four and 12 weeks of monitoring the flasks, no colonies could be detected on any medium. After 6 weeks of monitoring, five chocolate blood agar plates showed bacterial growth. In 75.0% of the samples, the bacterial species could be identified by using MALDI‐TOF. The bacterial species were identified as *Staphylococcus warnerii*, *Staphylococcus epidermidis*, *Staphylococcus pasteuri*, *Ralstonia insidiosa*, and *Alloiococcus otitidis*. After 8 weeks of monitoring the flasks, colonies of *Streptococcus oralis* could be detected on a further blood agar plate. All the other samples remained negative. In total, 1.5% of all culture media (*N* = 400), which corresponds to six out of the 50 samples (12.0%), showed a transitional bacterial contamination (Table 1). At all times, no fungi were detected. All positive results originated from different flasks.

**TABLE 1:** Positive agar probes, Roman numerals represent adhesive flask of sample origin numbered from I to X

Culture medium	Week of sample collection					Microorganism identified
	2	4	6	8	12	
Blood agar	—	—	—	IX	—	*Streptococcus oralis*
Chocolate blood agar	—	—	V, VI, VII, VIII, IX	—	—	*Staphylococcus warnerii*, *Staphylococcus epidermis*, *Ralstona insidiosa*, *Alloiococcus otitidis*, *Staphylococcus pasteuri*
Endoagar	—	—	—	—	—	
Sabaroudagar	—	—	—	—	—	
BHI boullion	—	—	—	—	—	
Thioglycolat boullion	—	—	—	—	—	
Columbiaagar, vitamin K, haemin	—	—	—	—	—	
Columbiaagar, vitamin K, haemin, gentamicin	—	—	—	—	—	

Abbreviation: BHI, Brain Heart Infusion.

## 4 DISCUSSION

The results of this longitudinal observation indicated a marginal transitional bacterial contamination of the dental tray adhesive in daily dental routine. Therefore, the null hypothesis was rejected. This observation is corroborated by other literature sources (Bensel et al., 2013; Herman, 1993). In contrast to these studies, the present investigation was not limited to a single bacterial species. The design of the study allowed the identification of a wide range of bacteria and fungi species that potentially survive the application of a disinfectant, and could consequently be transferred to another host. The strengths of the current study include the wide variety of culture mediums and the sophisticated microbiological methods for their identification. Unintended contamination of the agar plates or false positive results could be eliminated since a quality control in the manufacturing process of the plates was carried out and a diversity of species were observed. A limitation of the present investigation might be seen in the design of the sample collection, as it was not possible to identify the time of contamination or to quantify the contamination. Furthermore, the applied method was unsuited to identify viruses. Adding another test methodology could empower to evaluate the transmission risk of viruses like Hepatitis C, Herpes simplex or HIV. However, the chosen methodological approach was able to identify living microorganisms like *Staphylococcus warneri*, *Staphylococcus epidermidis*, *Staphylococcus pasteuri*, *Ralstonia insidiosa*, *Alloiococcus otitidis*, *and Streptococcus oralis*. These bacteria can be present in oral and epidermal loci and of are of special interest, since they are described as potentially pathogenic bacteria and components of biofilms (Weiland‐Brauer et al., 2019). Moreover, they are associated with infections throughout immunodeficiency (Savini et al., 2009) or ear diseases (Jervis‐Bardy et al., 2017).

In a most recent study growth of *Staphylococcus aureus*, *Pseudomonas aeruginosa*, *Escherichia coli*, and *Streptococcus mutans* could not be inhibited (Bensel et al., 2013). As mentioned beforehand, this is most probable due to early evaporation of the disinfectant. However, *Staphylococcus aureus, Salmonella choleraesuis*, and *Pseudomonas aeruginosa* were tested in another study, and all three bacteria showed relevant growth inhibition by dental tray adhesives (Herman, 1993). Due to the clinically oriented study design a second test verifying the contaminations was not feasible.

## 5 CONCLUSIONS

The disinfectant component of the tested tray adhesive containing isopropanol appeared to be bactericide, since detected contaminations were no longer observed during follow‐up probing. However, a basic risk of transmission of oral bacteria by tray adhesives with reusable brushes could be observed. Due to the small percentage of positive samples and the temporary contamination of the flasks, the risk of transmission of a relevant number of oral bacteria from patient to patient seems minimal. However, periods between point of contamination and probing could not be addressed in the applied study design.

## AUTHOR CONTRIBUTIONS

All authors contributed to the study conception and design. Material preparation and data collection were performed by Henrik Müller under supervision of Catalina Suzana Stingu. Study design and draft of the manuscript were written by Oliver Schierz. Sebastian Hahnel and Angelika Rauch supported the study realization and improved the manuscript. All authors commented on previous versions of the manuscript. All authors read and approved the final manuscript.

## CONFLICT OF INTERESTS

The authors declare no competing interest.

## ETHICS STATEMENT

This article does not contain any studies with human participants or animals performed by any of the authors.

## INFORMED CONSENT

For this type of study, formal consent is not required. The authors have no conflict of interest and no funding to declare.

## Data Availability

All data available are presented within the publication.
